# Relationship between *ALDH2* genotype and in-stent restenosis in Chinese Han patients after percutaneous coronary intervention

**DOI:** 10.1186/s12872-019-1161-9

**Published:** 2019-07-25

**Authors:** Lizhi Lv, Weijie Ye, Peiyuan Song, Yubin Chen, Jing Yang, Congmin Zhang, Xiaoping Chen, Fanyan Luo

**Affiliations:** 1 Department of Cardiothoracic Surgery, Xiangya Hospital, Central South University, Changsha, Hunan 410008 People’s Republic of China; 2 Department of Clinical Pharmacology, Xiangya Hospital, Central South University, Changsha, 410008 People’s Republic of China; 3 Department of Pharmacy, The First Affiliated Hospital of Zhengzhou University, Zhengzhou, 450052 Henan People’s Republic of China

**Keywords:** *ALDH2*, SNP, In-stent restenosis, Percutaneous coronary intervention, Genetic polymorphisms

## Abstract

**Background:**

It is well known that the genotype of *ALDH2* is associated with coronary artery disease (CAD), and in-stent restenosis (ISR) is a primary complication of percutaneous coronary intervention (PCI), a primary recommended treatment for CAD. The aim of this study was to identify the relationship between aldehyde dehydrogenase 2 (*ALDH2*) genotype and in-stent restenosis (ISR).

**Methods:**

This study recruited 531 patients who were undergoing PCI at two Chinese hospitals from 2015 to 2017 and 183 were diagnosed with ISR after PCI during the one-year follow-up period. We used polymerase chain restriction fragment length polymorphism (PCR-RFLP) and sequencing to determine *ALDH2* polymorphisms.

**Results:**

Among all 531 patients (mean age = 59.4 ± 9.8; 65.9% male), 68.7% carried the wild-type genotype, 28.4% were heterozygous for the mutation, and 2.8% were homozygous for the mutation. Multiple logistical regression analyses indicated no correlation between *ALDH2* genotype and the occurrence of restenosis after PCI (OR = 1.448, 95% CI: 0.965–2.168, *p* = 0.073), though a significant association was observed for patients with diabetes (OR = 4.053, 95% CI: 1.668–10.449, *p* = 0.003).

**Conclusion:**

In this study, we found that carrying an ALDH2*2 allele had no notable relationship with ISR one year after PCI but that it did have a significant association with complications in diabetic patients. Further studies with larger sample sizes will be necessary to reveal a consensus.

**Electronic supplementary material:**

The online version of this article (10.1186/s12872-019-1161-9) contains supplementary material, which is available to authorized users.

## Background

Coronary artery disease (CAD) is one of the most common causes of death worldwide, with over 7 million deaths each year [1]. According to the WHO, in 2011, China has the second-largest number of CAD deaths in the world. CAD is a complex disease with both genetic and environmental components, with genetic factors accounting for 40–50% of the etiology of CAD [2]. Treatment for CAD depends on drug therapy and percutaneous coronary intervention (PCI). As the recommended therapy when symptoms persist despite drug treatment, PCI is important not only in acute coronary syndrome (ACS) but also in stable CAD. Although application of PCI has greatly improved ACS patient prognosis, prolonged life and enhanced quality of life, some patients suffer from in-stent restenosis (ISR), which can lead to relapse and a poor prognosis [3]. Thus, ISR has become a common problem for PCI.

Understanding the mechanisms involved in the development of ISR is crucial for its prevention. The direct cause of ISR is stent-induced endothelial injury. Delayed reendothelialization in the stented segment can result in activation of cytokines and growth factors, which in turn stimulate vascular smooth muscle cell (VSMC) differentiation, migration and proliferation [4], followed by narrowing of the coronary lumen [5]. Moreover, oxidative stress promotes the reactivity of aldehydes, such as acetaldehyde, malondialdehyde (MDA) and 4-hydroxynonenal (4-HNE), further aggravating endothelial dysfunction. Human mitochondrial aldehyde dehydrogenase 2 (encoded by the *ALDH2* gene) is a key enzyme that metabolizes and eliminates 4-HNE [6]. Previous studies have indicated that mice with ALDH2 functional defects exhibit increased sensitivity to externally induced oxidative stress [7] and that inhibition of ALDH2 by oxidative stress is significantly associated with cardiac dysfunction in diabetic rats [8]. Therefore, ALDH2 is considered to be a marker and protector against oxidative stress [9], and deletion of the gene can increase oxidative stress.

There is evidence that genetic factors such as single-nucleotide polymorphism in the genes encoding human vascular endothelial cell growth factor (*VEGF)* and endothelial nitric oxide synthase (*eNOS)* are related to a risk of ISR [10], though it remains unknown whether polymorphisms in genes involved in ROS metabolism are also associated with this risk. The human *ALDH2* gene is located on chromosome 12 (12q24.2), and the loss-of-function polymorphism rs671 (Glu504Lys or ALDH2*2) in *ALDH2* decreases ALDH2 activity by approximately 90% [11]. Due to the important role of *ALDH2* in 4-HNE metabolism and in the 4-HNE-mediated oxidative stress in endothelial dysfunction, we speculate the ALDH2*2 polymorphism might affect the risk of ISR after PCI by influencing vascular endothelial function.

## Methods

### Study population

This study was conducted at Xiangya Hospital of Central South University and First Affiliated Hospital of Zhengzhou University. All patients were diagnosed with ACS and underwent PCI within 4 weeks after their diagnosis. All patients returned to the hospital for reexamination by coronary angiography about one year after discharge according to individual clinical practices. The inclusion criterion was a diagnosis of in-stent restenosis via coronary angiography after PCI treatment during the one-year follow-up period. The exclusion criteria were as follows: (1) presence of severe heart valve disease; (2) history of thrombosis; (3) cerebrovascular accident in the 4 months before the procedure; (4) bleeding susceptibility; (5) malignant disease such as tumor; (6) platelet dysfunction; (7) acute or chronic inflammatory disease; (8) incomplete information. A 5-ml sample of peripheral blood was drawn from all patients into ethylenediaminetetraacetic acid (EDTA) tubes and at centrifuged at 3000 rpm for 10 min; the plasma and blood cells were separated and stored at − 20 °C. Genomic DNA was extracted from the blood cells. A questionnaire and case registration form were used to record in detail the reasons for readmission, basic information, clinical presentation, coronary angiograph results, the event of IRS, and the degree of restenosis. The time of all patients to reexamination the angiography was recorded and the time from PCI to reexamination were calculated. Smoker vs non-smoker was distinguished by no cigarette smoking for half a year; drinker vs non-drinker was distinguished by no alcohol drinking for half a year. Hypertension, hyperlipidemia and diabetes mellitus were defined according to European Society of Hypertension (ESH)/European Society of Cardiology (ESC) guidelines for the management of arterial hypertension [12], the third Report of The National Cholesterol Education Program (NCEP) [13] and American Diabetes Association criteria [14], respectively.

### Definition of in-stent restenosis

The definition of ISR was greater than 50% reduction in the luminal diameter within the stent, distal segments adjacent to the stent or the 5-mm proximal, as based on follow-up angiography [12]. In patients with multiple restenosis, the number of lesions were recorded. The results of the luminal narrowing and in-stent restenosis were confirmed by two experienced interventional cardiologists according to ESC guidelines for cardiovascular diagnoses.

### ALDH2*2 (Glu504Lys) polymorphism assay

DNA was extracted by phenol-chloroform extraction performed according to previously described protocols [15]. The polymerase chain restriction fragment length polymorphism (PCR-RFLP) method was employed for genotyping using the following primers: upstream, 5′- GATGTGGAGGTTGCAACGAG − 3′; downstream, 5′- CCTACAGGCCTTGGCGTATA − 3′. The PCR amplification system used 15 ng of genomic template and 10 μM primers with the following reaction conditions: 36 cycles of 94 °C for 45 s, 60 °C for 45 s, and 72 °C for 1 min, followed by extension at 72 °C for 10 min and storage at 4 °C. The PCR product (3 μL) was digested using the restriction endonuclease Eco57 I at 37 °C overnight and electrophoresed through a 1.5% agarose gel with ethidium bromide staining. The PCR products were sent to Sangon Biotech Company for sequencing. Data were analyzed with the use of Chromas version 2.33. Genomic sequences of *ALDH2* obtained from GenBank (NC_000012.12:111766887–111809985) were used as references.

### Statistical analysis

The t test and chi-square test were performed with SPSS 20.0 software, and logistic regression analysis was performed using R statistical software version 3.3.3. Measurement data are expressed as the mean ± standard deviation and median (interquartile range). Analysis of continuous variables such as biochemical tests was performed using the t test and non-normally distributed data was performed using Mann-Whitney test or Kruskal-Wallis H test. Analysis of categorical variables such as sex, risk factors, coronary artery lesions, medications and *ALDH2* genotype were analyzed by the chi-square test. Logistic regression analysis was applied to assess the relationship between genotype and ISR risk. The independent variables examined were based on previous studies [16, 17]. A *p* value less than 0.05 was considered to indicate statistical significance.

## Results

### Eligible patients and clinical characteristics between ISR and non-ISR patients

**Fig. 1:**
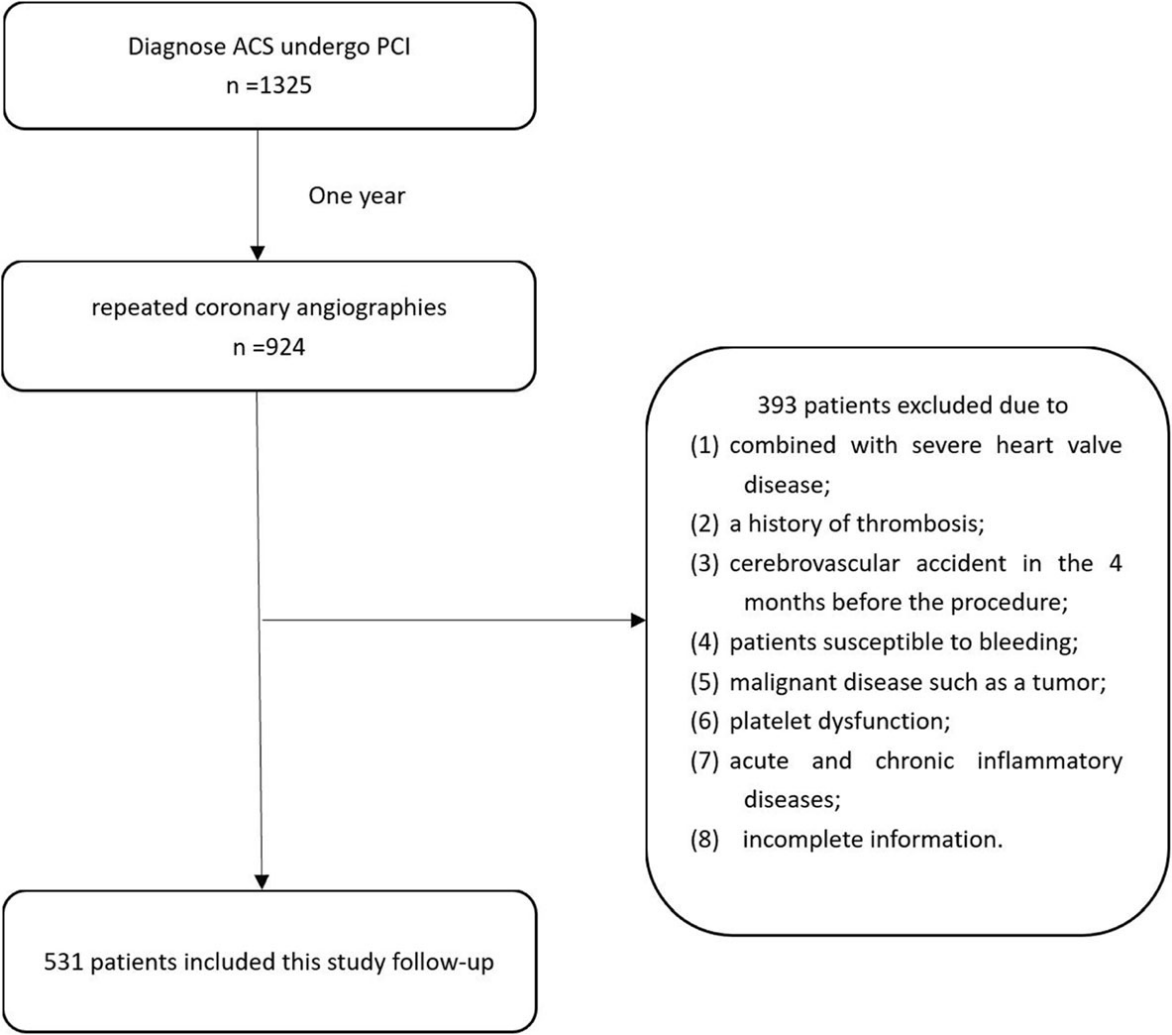
Patients selection flowchart

According to our inclusion and exclusion criteria, 531 subjects were enrolled in this study from 2015 to 2018 (Fig. 1). All of the patients visited the hospital and were subjected to repeated coronary angiographies after PCI. ISR was observed in 183 (34.5%) patients. The clinical characteristics of the patients enrolled in this study are listed in Table 1. Baseline demographics were balanced between the ISR group and the non-ISR group. Compared with non-ISR patients, ISR patients had higher HBA1C levels but lower thrombocytocrit levels. The incidence of type 2 diabetes and the use of clopidogrel and statins were notably overrepresented in patients with ISR. However, no differences in age, sex, follow-up period, risk factors, biochemical tests, or other medical treatments were found between the patients with and without ISR. Despite similarities with regard to the site of coronary lesion and degree of stenosis before PCI, patients with complicated coronary artery disease and multivessel disease were found to be more likely to develop ISR (Table 1).

**Table 1:** Baseline characteristics of patients who undergoing PCI

Variable	Total (*n* = 531)	ISR (*n* = 183)	no-ISR (*n* = 348)	*p*-value
Age, years	59.4 ± 9.8	58.8 ± 9.8	60.5 ± 9.8	0.069
Male, n (%)	350 (65.9)	114 (62.3)	236 (67.4)	0.202
Follow-up period, days	363 (289–386)	364 (294–391)	363 (285–385)	0.471
Risk factors, n (%)				
Cigarette smoking	143 (26.9)	42 (23)	101 (29)	0.134
Alcohol drinking	96 (18.1)	29 (15.8)	67 (19.3)	0.332
Hypertension	247 (46.5)	92 (50.3)	155 (44.5)	0.208
Diabetes mellitus	115 (21.7)	49 (26.8)	66 (19.0)	0.038
Hyperlipemia	31 (5.8)	6 (3.3)	25 (7.2)	0.068
Biochemical tests				
Thrombocytocrit, mmol/L	0.19 ± 0.07	0.18 ± 0.04	0.19 ± 0.08	0.041
MPV, fl	9.03 ± 3.0	8.85 ± 1.7	9.12 ± 3.5	0.338
HBA1C, %	6.23 ± 1.9	6.53 ± 1.9	6.07 ± 1.9	0.007
Glucose, mmol/L	5.43 ± 1.9	5.6 ± 1.9	5.35 ± 1.9	0.16
Serum creatinine, μmol/L	75.7 ± 35.2	71.8 ± 22.4	77.8 ± 40.2	0.06
TC, mmol/L	3.82 ± 1.1	3.79 ± 1.1	3.83 ± 1.2	0.673
TG, mmol/L	1.62 ± 1.1	1.59 ± 0.8	1.64 ± 1.2	0.646
HDL, mmol/L	1.07 ± 0.3	1.07 ± 0.3	1.07 ± 0.3	0.997
LDL, mmol/L	2.3 ± 0.9	2.3 ± 0.9	2.29 ± 0.9	0.895
Coronary artery lesions, n (%)				< 0.001
Single-vessel disease	225 (42.4)	64 (35)	161 (46.3)	
Double-vessel disease	168 (31.6)	49 (26.8)	119 (34.2)	
Triple-vessel disease	138 (26)	70 (38.3)	68 (49.3)	
Medications, n (%)				
Clopidogrel	420 (79.1)	156 (85.2)	264 (75.9)	0.011
Ticagrelor	119 (22.4)	37 (20.2)	82 (23.6)	0.38
Statins	505 (95.1)	179 (97.8)	326 (93.7)	0.036
ACEI/ARB	196 (37)	75 (41)	121 (34.9)	0.166
β-blocker	383 (72.1)	133 (72.7)	250 (71.8)	0.838
CCB	158 (29.9)	58 (32)	100 (28.8)	0.442
Antidiabetic therapy	264 (49.7)	99 (54.1)	165 (47.4)	0.143
Nitrate	520 (97.9)	181 (98.9)	339 (97.4)	0.251
*ALDH2* genotype, n (%)				0.028
ALDH2 *1/*1	365 (68.7)	115 (62.8)	250 (71.8)	
ALDH2 *1/*2	151 (28.4)	59 (32.2)	92 (26.4)	
ALDH2 *2/*2	15 (2.8)	9 (4.9)	6 (1.7)	

Note: This statistical analysis was performed by SPSS 20.0 software. *MPV* Mean Platelet Volume, *HBA1C* Hemoglobin A1c, *TC* Total Cholesterol, *TG* Triglyceride, *HDL* High Density Lipoprotein, *LDL* Low Density Lipoprotein, *ACEI* Angiotensin-Converting Enzyme Inhibitors, *ARB* Angiotensin Receptor Blocker, *CCB* Calcium channel blocker

### Genotype distribution of the ALDH2*2 polymorphism in ISR and non-ISR patients

**Fig. 2:**
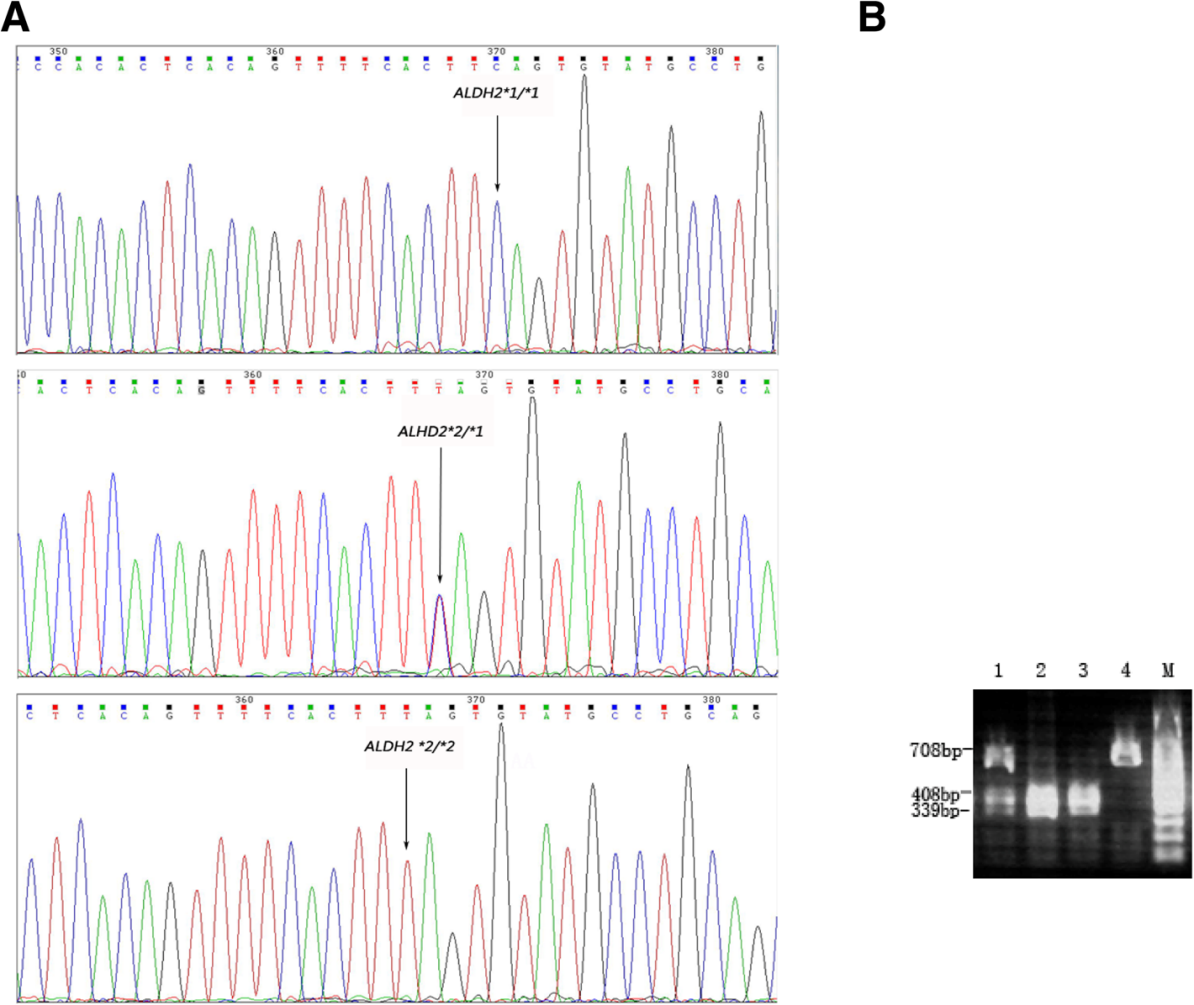
Genotyping Results of the *ALDH2*. Panel **a** was the results of the measure sequence: wild genotype of ALDH2 *1/*1, heterozygote genotype of ALDH2 *1/*2, homozygous mutant genotype of ALDH2 *2/*2. Panel **b** was the results of the *ALDH2* by PCR-RFLP: Sample 1 represents the heterozygote genotype of ALDH2 *1/*2, sample 2 and sample 3 represents the wild genotype of ALDH2 *1/*1, sample 4 represents the homozygous mutant genotype of ALDH2 *2/*2, sample M represents marker

The genotype distribution of the ALDH2*2 polymorphism is shown in Fig. 2. Among the 531 patients, 365 (68.7%) were the wild-type allele (ALDH2*1/*1), 151 (28.4%) carried the heterozygous (ALDH2*1/*2) allele and 15(2.8%)carried the mutant homozygote allele (ALDH2*2/*2). As the ALDH2*2 polymorphism is reportedly dominant, carriers of ALDH2*2 were combined for the ensuing association analysis [2]. As shown in Table 1, carriers of the ALDH2*2 allele were notably overrepresented among the ISR patients(*p* = 0.028).

### Association of the ALDH2*2 polymorphism with ISR risk

**Fig. 3:**
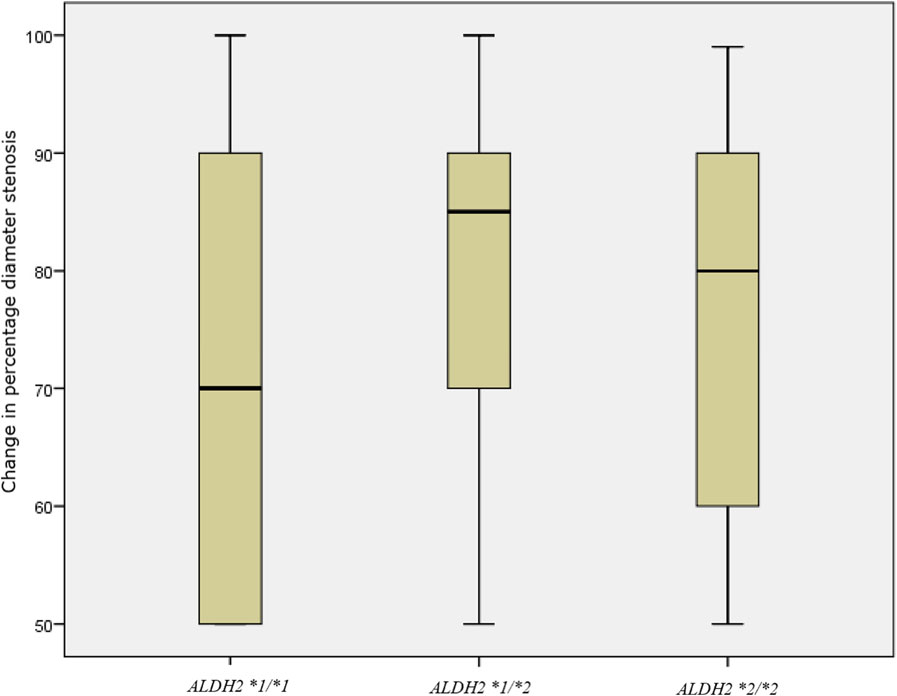
Correlation of genotype distribution and changes in percentage diameter stenosis. Correlation of genotype distribution and changes in percentage diameter stenosis was analyzed in 183 patients diagnosed with ISR. Among them, the median (interquartile range) of ALDH2 *1/*1 was 70 (50–90) %, ALDH2 *1/*2 was 85 (70–90) % and ALDH2 *2/*2 was 80 (60–92.5) %. Kruskal-Wallis H test was used to analyze the correlation, the result suggested that there was a statistically significant difference in the percentage diameter stenosis between the three groups (*p* = 0.034)

The results of the chi-square test revealed obvious difference in the prevalence of diabetes mellitus (*p* = 0.038), coronary artery lesions (*p* < 0.01) and the use of clopidogrel (*p* = 0.011) and statins (*p* = 0.036) between the ISR and non-ISR patients. Due to the low frequency of the mutant allele, heterozygotes with minor allele homozygotes were combined for comparison with wild-type allele homozygotes. Ten variables, including age, alcohol drinking, cigarette smoking, diabetes mellitus, coronary artery lesions, *ALDH2* genotype and medications (clopidogrel, statins, ACEI/ARB, and nitrite) were selected for univariate regression, and the results showed that patients with type 2 diabetes (OR = 1.647, 95% CI: 1.029–2.611, *p* = 0.035), carriers of the ALDH2*2 allele (OR = 1.537, 95% CI: 1.005–2.339, *p* = 0.046) and those who use of the antiplatelet medication clopidogrel (OR = 1.697, 95% CI: 1.018–2.934, *p* = 0.049) were at an increased risk for ISR (Table 2). In multivariate analysis, a significant association between coronary artery lesions (OR = 1.508, 95% CI: 1.193–1.911, *p* = 0.006, Table 2) and ISR was observed, and the use of clopidogrel for antiplatelet therapy was also a risk factor for ISR (OR = 1.680, 95% CI: 1.035–2.794, *p* = 0.039); in contrast, the association of *ALDH2* genotype with the risk of IRS was only marginal (OR = 1.50, 95% CI: 0.96–2.32, *p* = 0.073). Among the 183 patients diagnosed with ISR, the median (interquartile range) of ALDH2 *1/*1 was 70 (50–90) %, ALDH2 *1/*2 was 85 (70–90) % and ALDH2 *2/*2 was 80 (60–92.5) %. Analysis of correlation was performed using the Kruskal-Wallis H test and the result suggested that there was a statistically significant difference in the percentage diameter stenosis between the three groups (*p* = 0.034, Fig. 3).

**Table 2:** Risk Factors of in-sent retenosis by univariate and multivariate regression in all patients

Variable	Univariate analysis		Multivariate analysis	
	OR (95%)	*p*-value	OR (95%)	*p*-value
Age	1.01 (0.989–1.030)	0.378	1.009 (0.990–1.030)	0.32
Alcohol drinking (vs non)	0.877 (0.512–1.460)	0.622	1.230 (0.651–2.319)	0.521
Cigarette smoking (vs non)	0.807 (0.506–1.264)	0.357	0.654 (0.377–1.113)	0.123
Diabetes mellitus (vs non)	1.647 (1.029–2.611)	0.035	1.424 (0.909–2.221)	0.12
Coronary artery lesions (vs single-vessel disease)	1.278 (0.994–1.642)	0.057	1.508 (1.193–1.911)	0.006
ALDH2 *2 carriers vs ALDH2 *1 homozygotes	1.537 (1.005–2.339)	0.046	1.448 (0.965–2.168)	0.073
Clopidogrel	1.697 (1.018–2.934)	0.049	1.680 (1.035–2.794)	0.039
Statins	2.924 (0.992–12.503)	0.085	2.493 (0.867–9.054)	0.118
ACEI/ARB	1.317 (0.872–1.982)	0.188	1.052 (0.709–1.556)	0.799
Nitrite	1.739 (0.441–11.505)	0.483	1.367 (0.277–9.903)	0.718

Note: This statistical analysis was performed by R statistical software version 3.3.3. *ACEI* Angiotensin-Converting Enzyme Inhibitors, *ARB* Angiotensin Receptor Blocker

### Association of the ALDH2*2 polymorphism with ISR risk stratified by diabetes

Among ISR patients with diabetes, 27 (55.1%) carried the ALDH2*1/*1, 20 (40.8%) carried the ALDH2*1/*2 allele, and 2 (4.1%) carried the ALDH2*2/*2 allele (Table 3). Of the non-ISR patients with diabetes, 54 (81.8%) carried the ALDH2*1/*1 allele, 11 (16.7%) carried the ALDH2*1/*2 allele, and 1 (1.5%) carried the ALDH2*2/*2 allele (Table 3). The results of the chi-square test showed significant overrepresentation of ALDH2*2 carriers among ISR patients with diabetes (*p* = 0.008). Of the ISR patients who did not have diabetes, equal proportions of ALDH2*1/*1, ALDH2*1/*2 and ALDH2*2/*2 were 88 (65.7%), 39 (29.1%), and 7 (5.2%), respectively. No difference in *ALDH2* genotype distribution between the ISR and non-ISR patients was observed among those without diabetes (*p* = 0.139, Table 3).

**Table 3:** The genotype of *ALDH2* distribution in patients with or without diabetes mellitus

Genotype, n (%)	Total	ISR	non-ISR	*p*-value
Patients with diabetes				0.008
ALDH2 *1/*1	81 (70.4)	27 (55.1)	54 (81.8)	
ALDH2 *1/*2	31 (27)	20 (40.8)	11 (16.7)	
ALDH2 *2/*2	3 (2.6)	2 (4.1)	1 (1.5)	
Patients without diabetes				0.139
ALDH2 *1/*1	284 (68.3)	88 (65.7)	196 (69.5)	
ALDH2 *1/*2	120 (28.8)	39 (29.1)	81 (28.7)	
ALDH2 *2/*2	12 (2.9)	7 (5.2)	5 (1.8)	

Note: This statistical analysis was performed by SPSS 20.0 software

To investigate whether the occurrence of diabetes affects the relationship between the ALDH2*2 polymorphism and ISR risk, an additional association analysis was performed by stratification of patients with or without diabetes mellitus. In Table 4, 8 variables including age, alcohol consumption, cigarette smoking, coronary artery lesions, *ALDH2* genotype, and clopidogrel, statin and ACEI/ARB use are shown with their *p* values from univariate regression; among these, only ALDH2*2 (OR = 3.667, 95% CI: 1.606–8.727, *p* = 0.002) genotype was associated with a significantly increased risk for IRS among patients with diabetes. After entering these 8 variables into the multivariate model, the results were the same as those of univariate regression, with only ALDH2*2 (OR = 4.053, 95% CI: 1.668–10.449, *p* = 0.003) carriers showing significantly increased risk. Similarly, univariate regression and multivariate regression were employed to assess risk factors in non-diabetic patients, with no association found between *ALDH2* genotype and ISR (Table 4). Interestingly, both univariate and multivariate regression analyses indicated that coronary artery lesions were not associated with the risk of ISR among diabetic patients (OR = 1.095, 95% CI: 0.68–1.767, *p* = 0.709; OR = 1.068, 95% CI: 0.626–1.818, *p* = 0.808); however, in non-diabetic patients, the number of coronary artery lesions was significantly associated with ISR in both univariate and multivariate regression analyses (OR = 1.746, 95% CI: 1.355–2.261, *p* < 0.01; OR = 1.677, 95% CI: 1.284–2.2, *p* < 0.01). Additionally, the use of clopidogrel was associated with ISR in patients without diabetes (OR = 1.918, 95% CI: 1.129–3.382, *p* = 0.019; OR = 1.857, 95% CI: 1.073–3.329, *p* = 0.031) but not in those with diabetes (OR = 1.472, 95% CI: 0.551–4.221, *p* = 0.451; OR = 0.899, 95% CI: 0.297–2.829, *p* = 0.851).

**Table 4:** Risk Factors of in-sent retenosis by univariate and multivariate regression in patients with or without diabetes mellitus

	Variable	univariate analysis		multivariate analysis	
		OR (95%)	*p*-value	OR (95%)	*p*-value
Diabetes	Age	1.018 (0.974–1.065)	0.431	1.014 (0.966–0.966)	0.574
	Alcohol drinking (vs non)	0.644 (0.164–2.183)	0.495	0.944 (0.178–4.66)	0.944
	Cigarette smoking (vs non)	0.636 (0.187–1.929)	0.439	0.436 (0.093–1.113)	0.267
	Coronary artery lesions (vs single-vessel disease)	1.095 (0.680–1.767)	0.709	1.068 (0.626–1.818)	0.808
	ALDH2 *2 carriers vs ALDH2 *1 homozygotes	3.667 (1.606–8.727)	0.002	4.053 (1.668–10.449)	0.003
	Clopidogrel	1.472 (0.551–4.221)	0.451	0.899 (0.297–2.829)	0.851
	Statins	1.500 (0.140–32.83)	0.743	1.817 (0.145–2.794)	0.651
	ACEI/ARB	1.376 (0.654–2.914)	0.400	1.205 (0.528–2.748)	0.656
Non-diabetes	Age	1.015 (0.995–1.037)	0.145	1.009 (0.987–1.031)	0.424
	Alcohol drinking (vs non)	0.868 (0.508–1.445)	0.591	1.177 (0.581–2.374)	0.649
	Cigarette smoking (vs non)	0.801 (0.505–1.253)	0.337	0.694 (0.379–1.241)	0.225
	Coronary artery lesions (vs single-vessel disease)	1.746 (1.355–2.261)	< 0.001	1.677 (1.284–2.200)	< 0.001
	ALDH2 *2 carriers vs ALDH2 *1 homozygotes	1.191 (0.766–1.841)	0.433	1.112 (0.693–1.774)	0.657
	Clopidogrel	1.918 (1.129–3.382)	0.019	1.857 (1.073–3.329)	0.031
	Statins	3.333 (1.117–14.329)	0.055	2.594 (0.839–11.376)	0.138
	ACEI/ARB	1.210 (0.785–1.855)	0.385	1.008 (0.635–1.586)	0.974

Note: This statistical analysis was performed by R statistical software version 3.3.3. *ACEI* Angiotensin-Converting Enzyme Inhibitors, *ARB* Angiotensin Receptor Blocker

## Discussion

This study analyzed the association between the ALDH2*2 polymorphism and risk for ISR one year after PCI. Previous studies have demonstrated that the rate of the ALDH2*2 polymorphism is approximately 30–50% in Asians, but the frequency worldwide is only approximately 8% [18]. Therefore, analysis of the clinical relevance of this polymorphism is important for Asian populations. Several previous studies have reported that the ALDH2*2 polymorphism is a risk factor for the course of CAD, especially in Asians [18]. For example, Yavari et al. found the ALDH2*2 polymorphism to be an independent genetic risk factor for CAD in the Han Chinese population [19]. In addition, *ALDH2*-deficient mice exhibit significantly increased production of mitochondrial ROS and endothelial dysfunction after prolonged treatment with acetaldehyde [20]. Indeed, oxidative stress due to the production of reactive oxygen species (ROS) and inflammation are key pathophysiological processes for the development of ISR and for in-stent atherosclerosis [21]. ROS can cause endothelial dysfunction and directly promote VSMC proliferation and migration by inducing inflammation. ROS can also promote the formation of lipid free radicals and lipid peroxides, which result in the formation of reactive aldehydes, such as acetaldehyde, MDA and 4-HNE, further aggravating endothelial dysfunction. PC12 cells deficient in *ALDH2* are highly sensitive to 4-HNE-induced oxidative damage and accumulate proteins that are modified by 4-HNE. Ethanol can reduce 4-HNE levels by activating *ALDH2* and thus shows cardioprotective effects during ischemia-reperfusion injury [22, 23]. Therefore, ALDH2 is considered to be a marker of and protector against oxidative stress, and defects in *ALDH2* can increase oxidative stress [24]. Asymmetric dimethylarginine (ADMA) is a newly discovered risk factor for cardiovascular disease that acts via inhibition of NO synthase and decreased NO production, which leads to vascular endothelial dysfunction. Evidence has shown that 4-HNE can inhibit expression of miR-21, a microRNA that suppresses expression of the ADMA-inactivating enzyme dimethylarginine dimethylaminohydrolase 1 (DDAH1). In addition 4-HNE can directly form an adduct with DDAH1, inhibiting DDAH1 activity and promoting endothelial dysfunction [25]. The ALDH2*2 polymorphism is associated with CAD susceptibility [15], and the mechanism of this effect is partially explained by decreased DDAH1 expression and increased ADMA levels in endothelial cells. The stent-induced endothelial injury and delayed reendothelialization by DES is critical for the development of ISR, and interindividual differences in reendothelialization capacity might influence the risk of ISR and the prognosis of CAD after PCI [26, 27].

In this study, we analyzed 531 CAD patients who had received PCI and were diagnosed with in-stent restenosis about one year after the procedure. Patients were grouped according to the occurrence of ISR. Frequency matching was also performed for all variables potentially associated with a risk of ISR to evaluate the pure impact of the *ALDH2* polymorphism. The overall frequency of heterozygosity for the polymorphism was 31.2% in this study, and this frequency was comparable with that of other studies [18]. Our results showed that there was a statistical difference in the changes in percentage diameter stenosis among the three genotypes of 183 patients diagnosed with ISR. But it was different from our expectation that the percentage diameter stenosis of ALDH2 *1/*2 was the highest, which may be caused by low frequency of ALDH2 *2/*2. We acknowledged this study’s limitation that there is no specific value degree of stenosis for those patients who had reduction in in-stent luminal diameter of less than 50%, and we could not acquire their angiographic images for technical reason. Furthermore, our results indicated that ALDH2*2 carriers were at an increased risk of ISR, especially among those with diabetes. Wang et al. observed that myocardial MDA contents and ROS levels were significantly increased in diabetic rats, which also showed reduced left ventricular ejection fraction and fractional shortening accompanied by decreased *ALDH2* expression and activity. We hypothesized that a diabetic status may result in increased oxidative stress, which may include an increase in endogenous aldehydes levels. Furthermore, the level of oxidative stress in ALDH2*2 patients may be increased, thus accounting for the delayed reendothelialization of target vessels after DES-induced endothelial injury and the increased risk for ISR after PCI. In addition, the risk of ISR is higher in the Asian population than in European and American populations, perhaps because ALDH2*2 is more commonly found in Asians than in other populations [28].

### Study limitation

There are several limitations of this study. First, the sample size was small. Moreover, for analyzing the changes in percentage diameter stenosis as a continuous variable, we had to exclude those patients with a reduction of less than 50% in in-stent luminal diameter, because the angiographic images were not re-accessible for a technical reason. A prospective study with a larger sample size is required to validate our findings. Second, given that the distribution of different gene mutations can vary depending on ethnicity, our results might not be repeatable in other ethnic populations. Third, data regarding BMI, stent type, and angiographic and echocardiographic information were not collected at baseline, though these data might have prognostic significance. Lastly, the major weakness of this study is the lack of systematic follow-up of all patients, which may have resulted in selection bias. Although we showed no association between *ALDH2* genotype and ISR, we cannot ensure that our eligible cohort is representative of the population due to the loss patients because of poor compliance and incomplete follow-up.

## Conclusion

Our findings indicate that ALDH2*2 (heterozygous genotype) was not associated with ISR one year after PCI. Although our study demonstrated that ALDH2*2/1/ALDH2*2/2 were not associated with ISR after PCI, the well-documented cardiovascular protection provided by the ALDH2*1/1 genotype is unquestionable. Furthermore, the prevalence of nonfunctional alleles in different ethnic populations should be considered to achieve a consensus regarding the characteristics of these genotypes [2].

## Additional file

Additional file 1: **Table S1**. Clinical information of all patients in this study. (XLSX 90 kb)

## Data Availability

All data generated or analyzed during this study are included in this published article Additional file 1.

## Abbreviations

4-HNE: 4-Hydroxynonenal
ACEI: Angiotensin-converting enzyme inhibitor
ACS: Acute coronary syndrome
ADMA: Asymmetric Dimethylarginine
ALDH2: Aldehyde dehydrogenase 2
ARB: Angiotensin receptor blocker
BMS: Bare metal stent
CAD: Coronary artery disease
CCB: Calcium channel blocker
DDAH1: Dimethylarginine Dimethylaminohydrolase 1
DES: Drug-eluting stent
EDTA: Ethylenediaminetetraacetic acid
eNOS: Endothelial nitric oxide synthase
HBA1C: Hemoglobin A1c
HDL: High-density lipoprotein
ISR: In-stent restenosis
LDL: Low-density lipoprotein
MDA: Malondialdehyde
MPV: Mean platelet volume
PCI: Percutaneous coronary intervention
ROS: Reactive oxygen species
SNP: Single-nucleotide polymorphism
TC: Total cholesterol
TG: Triglyceride
VEGF: Vascular endothelial cell growth factor
VSMC: Vascular smooth muscle cell
